# Characterizing Hypervelocity Impact (HVI)-Induced Pitting Damage Using Active Guided Ultrasonic Waves: *From Linear to Nonlinear*

**DOI:** 10.3390/ma10050547

**Published:** 2017-05-18

**Authors:** Menglong Liu, Kai Wang, Cliff J. Lissenden, Qiang Wang, Qingming Zhang, Renrong Long, Zhongqing Su, Fangsen Cui

**Affiliations:** 1 Institute of High Performance Computing, A*STAR, Singapore 138632, Singapore; liuml@ihpc.a-star.edu.sg (M.L.); cuifs@ihpc.a-star.edu.sg (F.C.); 2 Department of Mechanical Engineering, The Hong Kong Polytechnic University, Kowloon, Hong Kong, China; menglong.liu@connect.polyu.hk (M.L.); kai-qf.wang@connect.polyu.hk (K.W.); 3 Department of Engineering Science and Mechanics, The Pennsylvania State University, University Park, State College, PA 16802, USA; cjl9@psu.edu; 4 School of Automation, Nanjing University of Posts and Telecommunications, Nanjing 210023, China; wangqiang@njupt.edu.cn; 5 State Key Laboratory of Explosion Science and Technology, Beijing Institute of Technology, Beijing 100081, China; qmzhang@bit.edu.cn (Q.Z.); longrenrong@bit.edu.cn (R.L.)

**Keywords:** hypervelocity impact, ultrasonic guided waves, nonlinear, structural health monitoring, space structures

## Abstract

Hypervelocity impact (HVI), ubiquitous in low Earth orbit with an impacting velocity in excess of 1 km/s, poses an immense threat to the safety of orbiting spacecraft. Upon penetration of the outer shielding layer of a typical two-layer shielding system, the shattered projectile, together with the jetted materials of the outer shielding material, subsequently impinge the inner shielding layer, to which pitting damage is introduced. The pitting damage includes numerous craters and cracks disorderedly scattered over a wide region. Targeting the quantitative evaluation of this sort of damage (multitudinous damage within a singular inspection region), a characterization strategy, associating linear with nonlinear features of guided ultrasonic waves, is developed. Linear-wise, changes in the signal features in the time domain (e.g., time-of-flight and energy dissipation) are extracted, for detecting gross damage whose characteristic dimensions are comparable to the wavelength of the probing wave; nonlinear-wise, changes in the signal features in the frequency domain (e.g., second harmonic generation), which are proven to be more sensitive than their linear counterparts to small-scale damage, are explored to characterize HVI-induced pitting damage scattered in the inner layer. A numerical simulation, supplemented with experimental validation, quantitatively reveals the accumulation of nonlinearity of the guided waves when the waves traverse the pitting damage, based on which linear and nonlinear damage indices are proposed. A path-based rapid imaging algorithm, in conjunction with the use of the developed linear and nonlinear indices, is developed, whereby the HVI-induced pitting damage is characterized in images in terms of the probability of occurrence.

## 1. Introduction

Numerous man-made spacecraft (e.g., satellites, space stations, and shuttles) co-exist with innumerable meteoroids and orbital debris (MOD) particles in low Earth orbit. MOD particles travel at such high speeds (on the order of the first cosmic velocity) that even a small particle, upon impacting a spacecraft, can severely jeopardize the integrity of the spacecraft [1,2]. This sort of impact is commonly referred to as a “hypervelocity impact” (HVI), a scenario typically involving an impacting velocity in excess of 1 km/s. HVI is ubiquitous in space, as evidenced by numerous impact lesions seen on spacecraft after every flight mission. Addressing such significance, HVI has received a great deal of attention since the 1950s when humans began extensive exploration into outer space [3].

According to National Aeronautics and Space Administration (NASA), more than 20,000 pieces of MOD particles that are larger than 10 cm are known to exist in low Earth and geosynchronous orbits, along with over 500,000 particles sized 1–10 cm and tens of millions smaller than 1 cm [4]. Nowadays NASA and the U.S. Department of Defense have been cooperating to establish a Space Surveillance Network, aimed at tracking MOD particles bigger than 5 cm, via which the conjunction assessments and collision avoidance maneuvers can be implemented to counter objects included in the network and prevent HVI. However, such a network is unwieldy to monitor those MOD particles that are smaller than 5 cm, and the impact from any of them, though small in size, can functionally compromise the spacecraft performance, with possible catastrophic consequences. Representatively, in 1996, a French satellite was damaged by MOD particles originating from a French rocket that had exploded a decade earlier [5]. In 2007, China carried out an anti-satellite test and launched a missile to purposefully destroy a decommissioned weather satellite for removing the satellite from the orbit [5]. However, the 3000 new pieces of MOD particles consequently produced in that test have posed vast HVI risk to other spacecraft, arousing a great deal of concern from the public. In 2009 a satellite in the Iridium Communications Constellation System was struck by the debris generated from an exploded satellite which was abandoned by the then Soviet Union, and this satellite failed immediately [5]. Notably, this HVI event created approximately 2000 new pieces of trackable debris, threating other spacecraft in orbit. More recently, the European-built Cupola added to the International Space Station in 2010 was suspected to be impacted by a paint flake or a small metal fragment, leaving a 7 mm-diameter circular chip [6].

To minimize the HVI-associated hazard to orbiting spacecraft, a diversity of shielding mechanisms has been developed and installed on space assets, especially for those with a requirement of a long service period or with habitation of astronauts. Taking a typical two-layer Whipple Shield [7] as an example, after the perforation of the outer shielding layer by an MOD particle in HVI, the shattered MOD particles, together with parts of the jetted materials of the outer shielding layer, form a debris cloud that further impacts the inner structure behind the outer shielding layer, leading to numerous craters or direct penetration of the inner structure. It is therefore that a timely perception of an HVI event and consequent evaluation of HVI-induced damage in space assets, including impact central location and the maximum depth of the formed craters, are of vital importance and necessity. Effective and accurate evaluation can be conducive to implementation of remedial and repair actions.

Nevertheless, being significantly distinct from ordinary types of damage such as an orifice or a dent engendered in a low-velocity impact case, HVI-induced damage in the inner shielding layer presents unique and complex features: hundreds of small craters and cracks disorderedly clustered over a wide area, entailing novel detection methods. Amongst various candidate detection approaches, the guided-ultrasonic-wave (GUW)-based method has gained prominence [8,9,10,11]. Central to the increased use of GUW is its superior capacity of probing a large area promptly with only a few transducers, consuming low energy, accessing hidden components omni-directionally, as well as responding to various types of damage possession with high sensitivity [12,13,14]. Without a necessity to terminate the normal service of an inspected system and with the potential to be manipulated in an automatic manner, the GUW-based method has proven to be cost-effective in striking a compromise among resolution, detectability, practicality, and cost, corroborating the philosophy of in situ structural health monitoring (SHM).

Most GUW-based detection and monitoring methods have been developed in a linear regime, termed linear ultrasonics, in which responding GUW signals (upon probing GUW’s interaction with damage) are examined and the signal features are extracted at the same frequency as that of the probing GUW. Linear ultrasonics techniques, however, according to the Huygens principle, are only able to evaluate damage with a size comparable with the probing wavelength [15], thus showing limitations when used for the detection of the unique HVI-induced pitting damage. The probing GUW may surpass most of the small-dimension individual damage without any evident linear features to be evidenced in the perceived signals. On top of that, individual pitting craters or cracks with a relatively large size in a pitted region may possess a dimension comparable to the wavelength of the probing GUW, and each of them acts as an obstacle to scatter the probing GUW, leading to high complexity of captured signals, because of the mixing and overlapping of mutually-interfering waves scattered by the large number of craters and cracks in the pitted region.

In contrast, nonlinear ultrasonics, in which the responding GUW signals are explored in the frequencies other than the excitation frequency, has found their superb niche for SHM towards undersized damage [16,17]. The premise of the use of nonlinear ultrasonics is that damage, even when small in dimension, can introduce or augment the nonlinearity in the medium that can modulate probing GUW and generate nonlinear features into GUW signals in the frequency domain. These nonlinear GUW features have been proven to have higher sensitivity to the microstructure and micro-damage than the conventional linear GUW features [18]. Deng [19,20] is among those who first pointed out the condition of the accumulative nonlinear second harmonic wave along the propagation distance. De Lima [21] further analyzed the accumulation and oscillation of second harmonic, sum-, and difference-frequency components of the Lamb wave based on the normal mode expansion. Both concluded that, unlike the longitudinal wave in a bulk medium and the surface acoustic wave in a surface medium with unconditionally accumulative second harmonics, there are only a few accumulative mode pairs available under deliberately chosen frequencies and modes. The reason is that two conditions: (1) synchronism, or phase matching (the same phase velocity) and (2) non-zero power flux, are essential to guarantee an accumulative nonlinear guided wave. Lissenden [22], Liu [23], and Chillara [24] analyzed all the possible mode pairs (including shear horizontal wave) of accumulative second or even third harmonics with a displacement gradient-based formulation, to point out all the possible wave mode pairs to be used.

Up to this point, the characterization of HVI-induced pitting damage has yet to be well investigated. By analyzing how the microstructure and undersized damage influence the nonlinear stress-strain relationship, the correspondingly generated nonlinear ultrasonics can trace back to those nonlinear features, which are reflected by the material microstructure and undersized damage barely detectable with linear ultrasonics. Thus, the detection philosophy using nonlinear GUW techniques has ushered a novel avenue of using nonlinear ultrasonics to evaluate damage such as HVI-induced pitting damage in the shielding structures.

Addressing the imminent need of evaluation of HVI-induced damage by MOD particles and the lack of relevant research on the use of nonlinear GUWs for characterizing HVI-induced damage, this study is dedicated to evaluating HVI-induced pitting damage using linear/nonlinear features of GUWs. The commercial software Abaqus^®^/Explicit, together with a self-written vectorized user defined material subroutine (VUMAT) nonlinear material subroutine, is used to simulate the linear/nonlinear features of wave propagation in real three dimensional (3-D) structures. Compared to the predominant application of COMSOL^®^ to simplified two dimensional (2-D) scenarios by other researchers [25], the newly adopted approach is far more efficient in calculation and is thus expanded to a real 3-D case. Upon a correct understanding of the linear/nonlinear features of GUWs, both linear/nonlinear damage indices (*DI*s) are accordingly extracted to quantitatively image the HVI-induced damage using a path-based probability imaging algorithm.

This paper is organized as follows. Section 2 briefly introduces the 2-D analytical solution of the nonlinear GUWs and the incremental form of the nonlinear stress-strain relation to be embedded into the VUMAT user subroutine. Section 3 concerns both 2-D and 3-D numerical modeling based on Abaqus^®^/Explicit and VUMAT to quantitatively analyze the accumulation effect of nonlinear second harmonic waves in cases including phase matching and phase mis-matching. The HVI-induced damage is presented, and the related linear/nonlinear features of GUWs are extracted as *DI*s in Section 4. Finally, damage characterization is implemented in Section 5 based on the *DI*s using the reconstruction algorithm for probabilistic inspection of damage (RAPID), followed with the concluding remarks in Section 6.

## 2. Theory and Rationale: Nonlinear Guided Waves

The nonlinear elastic mechanics of a solid medium can represent the 3-D stress–strain relation with a second-order approximation as follows [26]

(1) $$\sigma_{ij}=\left(C_{ijkl}+1/2M_{ijklmn}\varepsilon_{mn}\right)\varepsilon_{kl},$$

where $\sigma_{ij}$ is the stress tensor, $\varepsilon_{mn}$ and $\varepsilon_{kl}$ are the strain tensors; $C_{ijkl}$ is the second-order elastic (SOE) tensor; and $M_{ijklmn}$ is a tensor associated with the material and geometric nonlinearities. If only the first term in the parenthesis, $C_{ijkl}$, is retained, Equation (1) will represent the linear elastic 3-D Hooke’s Law.

The tensor $M_{ijklmn}$ in Equation (1) can be expressed in terms of the notation by Landau and Lifshitz [27] as follows

(2) $$M_{ijklmn}=C_{ijklmn}+C_{ijln}\delta_{km}+C_{jnkl}\delta_{im}+C_{jlmn}\delta_{ik},$$

where

(3) $$\begin{array}{ll} C_{ijklmn}= & \frac{1}{2}(\;A_{\delta_{ik}}I_{jlmn}+\delta_{il}I_{jkmn}+\delta_{jk}I_{ilmn}+\delta_{jl}I_{ikmn})\\ & \;+2B\left(\delta_{ij}I_{klmn}+\delta_{kl}I_{mnij}+\delta_{mn}I_{ijkl}\right)+2C\delta_{ij}\delta_{kl}\delta_{mn}, \end{array}$$

In Equations (2) and (3), $C_{ijklmn}$ is the introduced third-order elastic (TOE) tensor due to material nonlinearity, while geometric nonlinearity is manifested in the last three terms of Equation (2). $\delta_{km}$ and those in similar forms with different subscripts are the Kronecker deltas; $I_{jlmn}$ and those in similar forms are the fourth-order identity tensors. As displayed in Equation (3), the material nonlinearity-related term, $C_{ijklmn}$, is directly related to three TOE constants *A*, *B* and *C*, which are the inherent properties of the material, to be measured experimentally [28].

To start with, the inner shielding layer can be simplified as a 2-D plane strain waveguide for wave propagation, in which four stress components $\sigma_{11},\sigma_{22},\;\sigma_{33},\;and\;\sigma_{12}$ and three strain components $\varepsilon_{11},\;\varepsilon_{22},\;and\;\varepsilon_{12}$ are reserved. In this way, the 2-D stress-strain relations for the nonlinear material can be easily deduced from the 3-D stress-strain relation in Equation (1).

**Figure 1:**
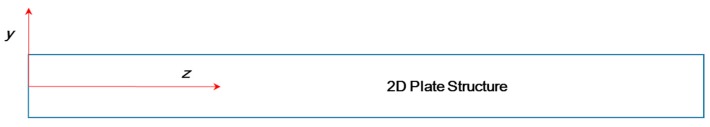
Stress-free 2-D plate structure.

If indices 1 and 2 in the Cartesian coordinate are projected to *y* and *z* as the thickness and propagation direction (see Figure 1), the amplitude of the second harmonic can be expressed as [21],

(4) $$A_{m}\left(z\right)={\bar{A}}_{m}\left(z\right)e^{i\left(k_{a}+k_{b}\right)z}-{\bar{A}}_{m}\left(0\right)e^{ik_{n}^{*}z},$$

where

(5) $${\bar{A}}_{m}\left(z\right)=\frac{i\left(f_{n}^{vol}+f_{n}^{surf}\right)}{4P_{mn}\left[k_{n}^{*}-\left(k_{a}+k_{b}\right)\right]},\;k_{n}^{*}\neq k_{a}+k_{b},$$

(6) $${\bar{A}}_{m}\left(z\right)=\frac{\left(f_{n}^{vol}+f_{n}^{surf}\right)}{4P_{mn}}z,\;k_{n}^{*}=k_{a}+k_{b},$$

where $\kappa_{a}$ and $\kappa_{b}$ denote the wave numbers of two fundamental modes. In all the cases studied in the paper, $\kappa_{a}=\kappa_{b}$, which gives the second harmonic (whose wave number is $\kappa_{m}$) twice the frequency as the fundamental mode. $\kappa_{n}$ is the wave number of the mode identified by the index *n* that is not orthogonal to the mode with wave number $\kappa_{m}$. $P_{mn}$ is the complex power flux in the direction $n_{z}$ of the mth propagating mode in the expansion of the secondary solution. It is calculated by the integral of the particle velocity multiplied by the stress tensor corresponding to the second harmonics over the entire waveguide cross section. The orthogonality relation for the propagating and evanescent modes is given as [29]

(7) $$P_{mn}=0,\;k_{m}\neq k_{n}^{*},$$

Thus a propagating mode *m* is orthogonal to all modes except itself, which makes *m* = *n*. In Equations (5) and (6), $f_{n}^{surf}$and $f_{n}^{vol}$ denote the power flux through the surface and through the volume, respectively, due to nonlinear terms of the primary wave. Both variables contain the square of the fundamental mode displacement as shown in [21]. Thus a relative acoustic nonlinearity parameter (RANP) $\beta^{\prime }$ is defined as

(8) $$\beta^{\prime }=\frac{A2}{A1^{2}},$$

where A2 and A1 denote the amplitude of the second harmonic and fundamental mode, respectively.

In the case of $\kappa_{n}\neq \kappa_{a}+\kappa_{b}$, *A*2 remains bounded and oscillates with a spatial periodicity, often called the dispersion length $L_{n}$, expressed as

(9) $$L_{n}=\frac{2\pi }{\left|k_{n}-2k_{f}\right|}\;,$$

where $\kappa_{f}$ denotes $\kappa_{a}$ or $\kappa_{b}$ ($\kappa_{a}=\kappa_{b}$ in all the studied cases), the wave number of the fundamental mode.

In the case of $\kappa_{n}=\kappa_{a}+\kappa_{b}$, the condition of phase matching is satisfied between the fundamental mode and specific mode in the expansion of the secondary solution. If the power flux is not zero ($f_{n}^{surf}+f_{n}^{vol}\neq 0$), *A*2 grows linearly along the propagation direction, which is called ‘internal resonance’. In conclusion, the internal resonance (linear growth of the second harmonic) requires two conditions, (1) phase matching and (2) non-zero power flux ($f_{n}^{surf}+f_{n}^{vol}\neq 0$).

Numerical simulation offers an alternative and a supplement to the analytical solution. An incremental form of the nonlinear stress-strain relation is deduced in this section. In real applications, an excitation signal has a finite time duration. Thus, the signal has a frequency spectrum of finite bandwidth instead of a single frequency that is assumed in the analytical analysis. In particular, the aforementioned analytical analysis resides on the 2-D plane strain assumption, while in real experiments, waves propagate in 3-D structures, in which the wave diffraction will exert an influence on the amplitude of waves.

The modeling platform Abaqus^®^/Explicit with the user defined VUMAT subroutine is adopted to realize the numerical modeling. Compared to Abaqus^®^/Standard and COMSOL^®^ that utilize an implicit algorithm, Abaqus^®^/Explicit is suitable for the transient wave propagation problem, with a controlled time increment to ensure the solving accuracy and stability. As the simulation is a process of transient dynamics in nature, the Forward Euler algorithm (explicit integration) is used to convert the constitutive rate equation, defined according to Equation (10), to an incremental equation. Abaqus^®^/Explicit provides a user subroutine block called VUMAT, which allows users to program the specified stress-strain relation into an incremental form. A two-state architecture is adopted where in each iteration variables including the strain increment and stress increment have their initial values in the old state and updated values in the new state. The updated stress in Equation (1) can be expressed as

(10) $$\sigma_{ij}+\Delta \sigma_{ij}=\left(C_{ijkl}+\frac{1}{2}M_{ijklmn}\left(\varepsilon_{mn}+\Delta \varepsilon_{mn}\right)\right)\left(\varepsilon_{kl}+\Delta \varepsilon_{kl}\right),$$

where Δε and Δσ are the strain and stress increments, respectively. Expanding the right side of Equation (10), the stress increment can be expressed as

(11) $$\begin{array}{ll} \Delta \sigma_{ij} & =(C_{ijkl}+\frac{1}{2}M_{ijklmn}\varepsilon_{mn}+\frac{1}{2}M_{ijklmn}\Delta \varepsilon_{mn})\left(\varepsilon_{kl}+\Delta \varepsilon_{kl}\right)-\left(C_{ijkl}+\frac{1}{2}M_{ijklmn}\varepsilon_{mn}\right)\varepsilon_{kl}\\ & =(C_{ijkl}+\frac{1}{2}M_{ijklmn}\Delta \varepsilon_{mn})\Delta \varepsilon_{kl}+\;M_{ijklmn}\varepsilon_{mn}\\ & =\left(C_{ijkl}+\frac{1}{2}M_{ijklmn}\left(\Delta \varepsilon_{mn}+2\varepsilon_{mn}\right)\right)\Delta \varepsilon_{kl}\;, \end{array}$$

Transforming Equation (11) into the VUMAT written in Fortran 77, the material and geometric nonlinearities are embedded into the constitutive relation. Based on the explicit integration, these variables are all vectorized to facilitate the computing process. In order to keep the simulation stability and accuracy, the time increment is controlled automatically by the global stable increment estimator in Abaqus^®^/Explicit. Moreover, considering that the higher-order harmonic components are relatively small, double precision and excitation of considerably large amplitudes are specified in simulation to ensure computation accuracy and excite finite-amplitude waves.

## 3. Numerical Simulation of Nonlinear Guided Waves

Both 2-D and 3-D numerical models with nonlinear stress-strain relations are built, featuring the frequently investigated S_0_-S_0_ and S_1_-S_2_ modes, to gain an insight into the accumulative nature of RANP.

### 3.1. 2-D Scenario

#### 3.1.1. Problem Description

**Figure 2:**
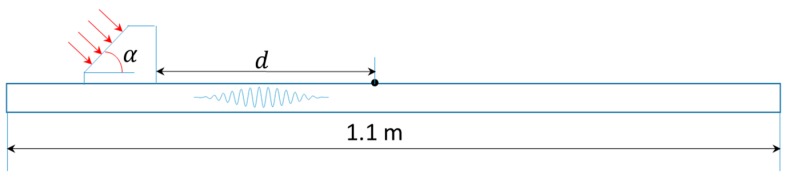
Schematic of 2-D plane strain simulation model for the mode pairs S_0_-S_0_ and S_1_-S_2_.

**Figure 3:**
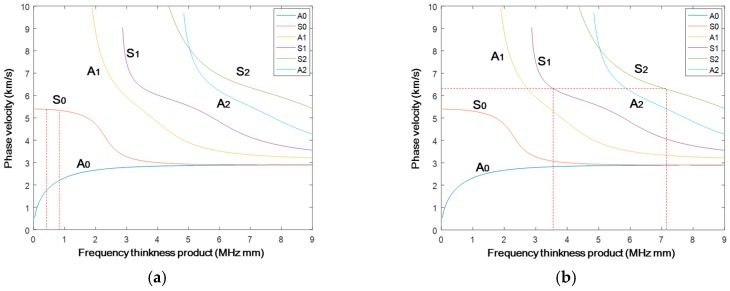
Phase velocities for the mode pair (**a**) S_0_-S_0_ 0.4–0.8 MHz mm and (**b**) S_1_-S_2_ at 3.534–7.068 MHz mm.

A plate with a thickness of 1 mm is modeled for the mode pair S_0_-S_0_, to which Plexiglas angle beam assemblies (see Table 1 for material parameters) are bonded in order to realistically reflect the mechanism of how an ultrasonic wave is excited into the plate structure (Figure 2). To model the linear/nonlinear features of wave propagation, the material parameters including density, Young’s modulus, Poisson’s ratio, and TOE constants listed in Table 1 [21] are defined as the input variables of VUMAT. A uniform displacement of twenty-cycle Hanning-window modulated sinusoidal toneburst at a central frequency of 400 kHz with the corresponding mode pair S_0_-S_0_ shown in Figure 3a, is introduced at the inclined plane of the wedge. This S_0_-S_0_ case of phase mis-matching (case 1 in Table 2) is deliberately set to quantitatively analyze the dispersion length. The incident angle of 30° is set according to Snell’s law [30]. Through a fixed coupling between the angle beam assemblies and the plate, the ultrasonic longitudinal vibration is converted to in-plane wave propagation. A fine mesh with the element length of 0.2 mm, 1/30 of the wavelength of the second harmonic S_0_ mode, is assigned to the whole model. As illustrated in Figure 2, measurement points are defined from *d* = 0 to 0.87 m at an interval of 10 mm for the acquisition of the in-plane displacement.

To model the mode pair S_1_-S_2_ with phase matching (case 2 in Table 2), a plate with a thickness of 3 mm is used. Based on the calculation using Snell’s law, the incident angle is adjusted to be 25° to ensure the in-plane propagation of the refracted S_1_ wave from the wedge into the plate. A uniform displacement of twenty-cycle Hanning-window modulated sinusoidal toneburst at a central frequency of 1.178 MHz (with corresponding mode pair S_1_-S_2_ of 3.534 MHz mm–7.068 MHz mm displayed in Figure 3b), is introduced at the inclined plane of the wedge. A fine mesh with the element length of 0.05 mm, 1/50 of the wavelength of the second harmonic S_2_ mode, is assigned to the whole model.

#### 3.1.2. Results and Discussion

**Figure 4:**
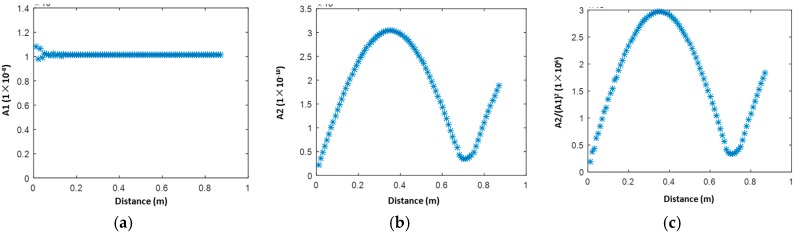
Magnitudes of the S_0_-S_0_ mode pair (0.4 MHz mm–0.8 MHz mm) extracted through Short-time Fourier Transform (STFT) in the 2-D simulation model: (**a**) fundamental mode A1; (**b**) second harmonic mode A2; and (**c**) A2/(A1)^2^.

The simulation with the excitation of the central frequency of 400 kHz is performed first. Short Time Fourier Transform (STFT) is applied to the extracted in-plane displacement, to obtain the wave energy packets corresponding to the fundamental and second harmonic modes. The maximum values of the first wave packets are extracted to indicate the amplitude of both modes. As plane waves propagate without wave diffraction, the fundamental S_0_ mode (Figure 4a) remains largely unchanged except at the near-field area. It is noticed that for the S_0_-S_0_ mode, the accumulation of the second harmonic S_0_ mode lasts up to a distance around 400 mm, making a dispersion length around 750 mm (corresponding to the valley in Figure 4b,c). An analytical solution of the dispersion length based on Equation (9) is given. In the current case, $\kappa_{n}=2w/c_{p-2w}$, and $\kappa_{a}=w/c_{p-w}$, which gives the dispersion length as

(12) $$L_{n}=\frac{1}{2f_{a}\left(\frac{1}{c_{p-2w}}-\frac{1}{c_{p-w}}\right)}=0.8584\;m.,$$

The obtained analytical result corresponds well to the numerical result (circa 750 mm), which validates the accuracy of the built numerical model. The rationale of the variation trend is that within 400 mm, the condition of phase matching can be satisfied approximately to reach the accumulative second harmonic S_0_ mode. However, after that distance, the second harmonic mode will gradually decrease in its amplitude to a valley as the phase mis-matching dominates. Then the second harmonic S_0_ mode re-accumulates since the phase matching is satisfied again.

**Figure 5:**
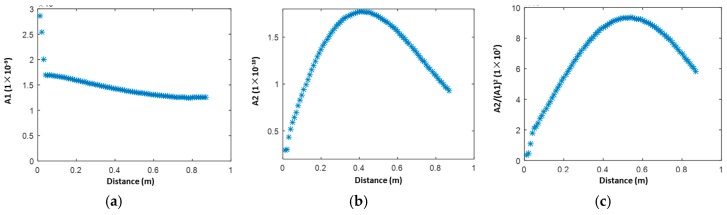
Magnitudes of the S_1_-S_2_ mode pair (3.534–7.068 MHz mm) extracted through STFT in the 2-D simulation model (**a**) fundamental mode A1; (**b**) second harmonic mode A2, and (**c**) A2/(A1)^2^.

The mode pair S_1_-S_2_ is also investigated following the same analysis procedure as the previous S_0_-S_0_ mode. A slight decrease of the S_1_ mode along the propagation distance is displayed in Figure 5a, which can be attributed to the severe dispersion of phase velocity for the S_1_ mode (see Figure 3b). As shown in Figure 5b, the second harmonic S_2_ mode shows a monotonic increase of amplitude over the propagation distance up to 400 mm. Taking the amplitude decrease of the fundamental mode (Figure 5a) into account, the calculated RANP indicates a monotonic and almost linear increase up to ~500 mm, which does not corroborate the analytical analysis of infinite accumulation of the second harmonic S_2_ mode according to Equation (6). The inconsistency, which is frequently observed in other studies as reviewed in [31], may arise from several aspects: (1) the selected central frequency may deviate from the frequency for precise phase matching of the S_1_-S_2_ mode; (2) the severe dispersion of phase velocity for the S_1_ mode may prevent the accumulation of the S_2_ mode to a long distance; (3) the ability of the current commercial Finite Element code to accurately capture the faint displacement. Nevertheless, a qualitative conclusion that the S_2_ mode accumulates along the propagation direction is achieved. In addition, among all these excited modes, S_1_ and S_2_ propagate with the fastest group velocity [32], which makes them superior to other mode pairs when dealing with material characterization or damage detection [32,33].

### 3.2. 3-D Scenario

All the above models are built in the assumption of 2-D plane strain, which, compared to the actual case of wave propagation in the 3-D plate structures, neglects the influence of diffraction. To quantitatively study the influence of diffraction, a 3-D model with only the mode pair S_0_-S_0_ is built. The 3-D S_1_-S_2_ model is not built here, considering more calculation effort is required for higher frequency.

#### 3.2.1. Problem Description

**Figure 6:**
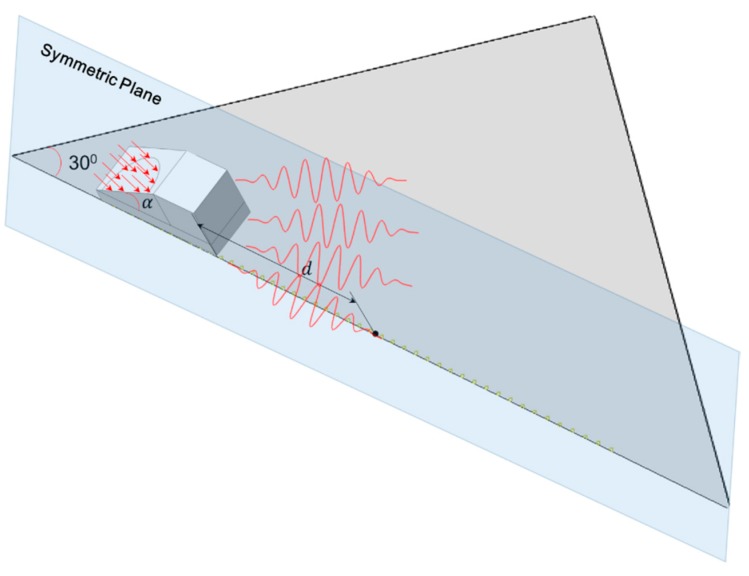
Schematic of the 3-D simulation model for the mode pair S_0_-S_0_.

In the 3-D model, the plate thickness of 1 mm and the excitation central frequency of 400 kHz are set (see Figure 6), the same as that in the 2-D S_0_-S_0_ model. Enormous calculation resources are consumed to model wave propagation in a 3-D structure. Two operations to reduce the model size are accordingly performed. Firstly, a symmetric boundary condition is applied to reduce the model to half of its original size. Secondly, the beam divergence angle of the fundamental mode, which is simulated to be less than 30°, enables a further reduction of the calculation domain through modeling of an isosceles triangle with a base angle of 30° in the in-plane dimension. A mesh size of 0.25 mm is assigned to the whole model, with approximately 26 nodes assigned in one wavelength of the concerned second harmonic wave mode S_0_, resulting in a calculation model comprising over 10 million elements.

#### 3.2.2. Results and Discussion

**Figure 7:**
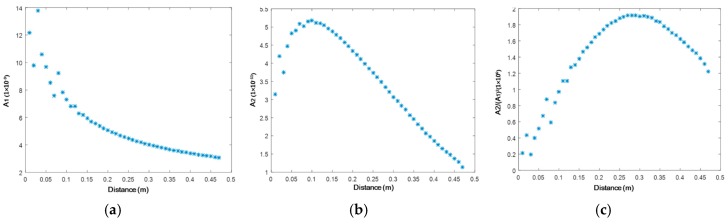
Magnitudes of the S_0_-S_0_ mode pair (0.4 MHz mm–0.8 MHz mm) extracted through STFT in the 3-D simulation model (**a**) fundamental mode A1; (**b**) second harmonic mode A2; and (**c**) A2/(A1)^2^.

The variation trend of magnitudes along the propagation distance (Figure 7) in the 3-D case is significantly different from that in the 2-D case (Figure 4). A drastic decrease in the magnitude of the fundamental mode occurs due to wave diffraction (Figure 7a). The magnitude of the second harmonic S_2_ mode only accumulates up to a propagation distance of 100 mm (Figure 7b), beyond which the diffraction dominates and suppresses the further accumulation of the second harmonic wave. However, RANP continues to accumulate up to 300 mm. Although the correction of diffraction based on the analytical 3-D nonlinear Raleigh wave is available [34], the analytical solution of the nonlinear Lamb wave taking the diffraction into account is yet to be developed. RANP deduced from the 2-D case (see Equation (8)) was used in most studies [32,33,35,36], which is also applied in this paper, while some works [37] tried to correct RANP for guided Lamb waves. Using RANP calculated from Equation (8), a qualitative agreement of RANP in the numerical 3-D case (Figure 7c) corresponds well with the 2-D case (Figure 4c).

## 4. Experimental Validation

Upon the establishment of the analytical and numerical foundation of linear/nonlinear GUWs with nonlinear stress-strain relation in the last two sections, the *DI*s based on linear/nonlinear GUWs are extracted to characterize HVI-induced pitting damage in the inner shielding layer. This kind of damage poses a threat to the structural integrity and leads to further potential injury to the equipment and crew inside.

### 4.1. HVI Experiment

**Figure 8:**
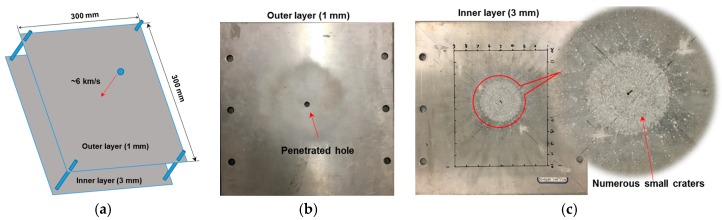
(**a**) Schematic of the Hypervelocity Impact (HVI) experiment; (**b**) photograph of the outer layer being penetrated; and (**c**) photograph of the inner layer with multitudinous small craters.

The HVI facilities at the State Key Laboratory of Explosion Science and Technology, China, were used for the HVI experiments, of which the core equipment is a two-stage light gas gun. Through the propulsion of gas of extremely high pressure, a projectile can be accelerated to impinge a target structure, at a desired velocity up to 10 km/s–the impact velocity in a typical HVI event in the low Earth orbit between a MOD particle and spacecraft. A two-layer shielding assembly is immobilized at the testing chamber of the two-stage light gas gun, which launches a projectile (aluminum 2024-T4, ~6 km/s, ø4.5 mm) into the thinner outer layer (aluminum 2024-T4, 1 mm in thickness, 300 mm × 300 mm in the in-plane dimension), to produce a controlled HVI (see Figure 8a). Upon penetrating the outer layer (see Figure 8b) using the vast instant kinetic energy, the shattered projectile, together with some jetted portion of the outer layer, further impacts the thicker inner layer (3 mm in the thickness). As shown in Figure 8c, multitudinous small-scale pitting damage is scattered over a large area, the center of which is filled with continuous craters, while the surrounding area is filled with separated craters.

#### 4.1.1. Experimental Setups

**Figure 9:**
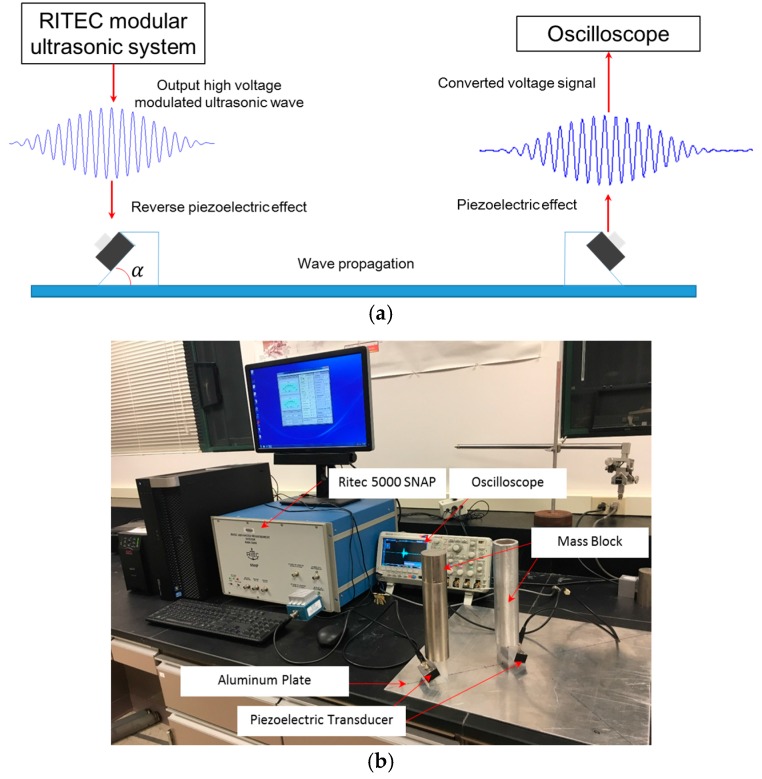
(**a**) Schematic of the experimental setup for the S_0_-S_0_ and S_1_-S_2_ mode pairs; and (**b**) photographic illustration of the experiment.

Figure 9 shows the schematic and photograph of the experiment. The RITEC advanced measurement system RAM-5000 SNAP (RITEC, Warwick, US) is used to generate a Hanning-window modulated ultrasonic voltage excitation around 1000 Vp-p. The piezoelectric transducer converts the electrical signal to the longitudinal ultrasonic vibration, which is then transmitted into a Plexiglas wedge with ultragel as the couplant. Based on Snell’s law, the vibration refracts and finally propagates in the in-plane direction of the plate structure through a further coupling between the angle beam assemblies and the plate. The actuator is fixed, and the receiver moves along the propagation direction. To suppress the measurement uncertainty, an average of 256 signals is applied to the acquired response voltage signal. Four repeated experiments are performed to verify the credibility of the measured data.

Experiments on both mode pairs S_0_-S_0_ and S_1_-S_2_ are performed. Regarding the mode pair S_0_-S_0_ 0.4 MHz mm–0.8 MHz mm for experiment validation, a 20-cycle Hanning-window modulated sinusoidal toneburst at a central frequency of 400 KHz is adopted as the excitation signal. Two piston piezoelectric transducers (KRAUTKRAMER^®^, ø25.4 mm) with respective central frequencies of 0.5 MHz and 1 MHz (as actuator and receiver, respectively) are coupled with an aluminum plate of 1 mm thickness. Water is applied as the couplant to ensure a consistent coupling as the location of the receiver changes. The second test is for the mode pair S_1_-S_2_ 3.42–6.84 MHz mm. A 20-cycle Hanning-window modulated sinusoidal toneburst at a central frequency of 1.14 MHz is adopted as the excitation signal. Two piston piezoelectric transducers (KRAUTKRAMER^®^, ø25.4 mm) with respective central frequency of 1 MHz and 2.25 MHz (as actuator and receiver, respectively) are coupled with an aluminum plate of 3 mm thickness. Honey is applied as the couplant to ensure a transfer of in-plane vibration from the angle beam assemblies to the aluminum plate structure.

#### 4.1.2. Results and Discussion

**Figure 10:**
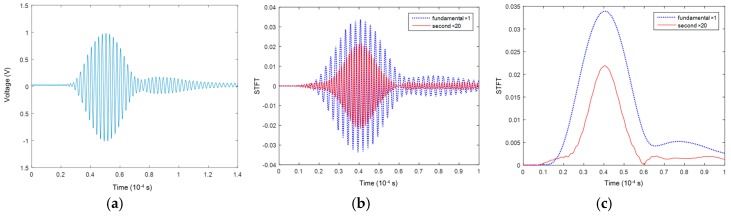
Signals of the mode pair S_0_-S_0_ at a propagation distance of 200 mm in the experiment (second harmonic mode × 20 times in amplitude) (**a**) raw acquired signals; (**b**) separated signals of S_0_-S_0_; and (**c**) separated energy packets of S_0_-S_0_.

Figure 10 displays the typical waveforms for the S_0_-S_0_ mode pair. Taking the raw time-domain signal acquired after a propagation distance of 200 mm (Figure 10a) as an example, the S_0_ mode is observed to occupy the dominant energy. The extracted magnitudes of the fundamental and second harmonic modes (Figure 10b,c) through STFT show that (1) the second harmonic mode is much weaker compared to the fundamental mode; and (2) the equal arrival time of the fundamental and second harmonic modes, the indicator of quasi-matching propagation velocities, guarantees the accumulation of the second harmonic mode.

**Figure 11:**
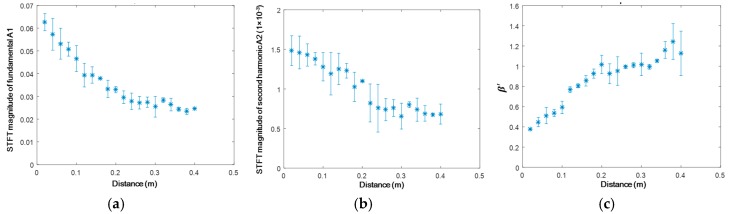
Magnitudes of the S_0_-S_0_ mode pair (0.4 MHz mm–0.8 MHz mm) extracted through STFT in four repeated experiments (**a**) fundamental mode A1; (**b**) second harmonic mode A2; and (**c**) A2/(A1)^2^.

The magnitude of the extracted wave energy packet is used as the index to quantitatively calculate RANP. Figure 11 shows the magnitudes of the extracted wave packet along the propagation distance for the S_0_-S_0_ mode pair. The average value and error bar calculated by the standard deviation are obtained according to four repeated experiments. Attributed to the wave diffraction, the fundamental mode (Figure 11a) decreases almost monotonically. The second harmonic mode (Figure 11b) decreases slowly within a distance of 0.16 m by the superposed influence of wave diffraction and nonlinear accumulation. RANP shows an approximately linear increase (Figure 11c), which corroborates both the analytical and numerical analyses in Section 2, Section 3.1, and Section 3.2.

**Figure 12:**
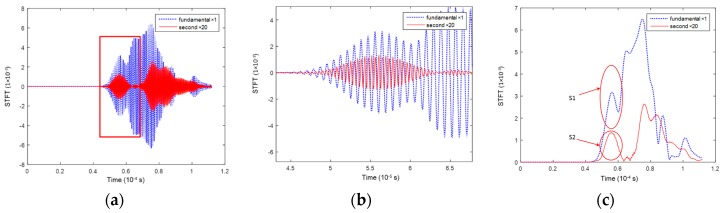
Signals of the mode pair S_1_-S_2_ 3.42 MHz mm–6.84 MHz mm at a propagation distance of 200 mm in the experiment (second harmonic mode × 20 times in amplitude). (**a**) Separated signals of the fundamental and second harmonic modes; (**b**) zoom-in signals of the S_1_ and S_2_ modes; and (**c**) separated energy packets of the S_1_ and S_2_ modes.

Figure 12 displays typical waveforms for the S_1_-S_2_ mode pair. The fundamental wave (Figure 12a) comprises multiple wave modes according to the dispersion curve in Figure 3b, in which the S_1_ mode (Figure 12b) arrives first, together with the S_2_ mode in the second harmonic component. Taking the signal acquired after a propagation distance of 200 mm (Figure 12) as an example, STFT analysis is performed to extract the wave packet of the fundamental S_1_ mode and second harmonic S_2_ mode (Figure 12c). S_1_ and S_2_ arrive the earliest among the multiple components of the wave modes, which facilitates the quantitative analysis on the accumulation effect of the S_2_ mode.

**Figure 13:**
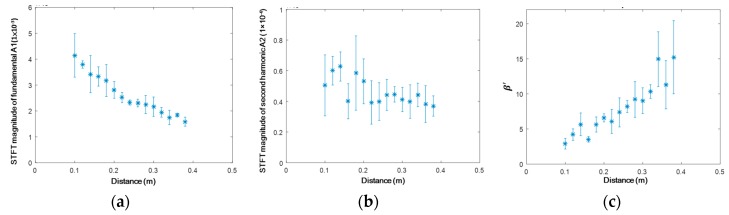
Magnitudes of the S_1_-S_2_ mode pair (3.42 MHz mm–6.84 MHz mm) extracted through STFT in four repeated experiments. (**a**) Fundamental mode A1; (**b**) second harmonic mode A2; and (**c**) A2/(A1)^2^.

The magnitude of the extracted wave energy packet (Figure 13) is used as the index to quantitatively calculate RANP. Owing to wave diffraction, the fundamental mode decreases almost monotonically (Figure 13a). The second harmonic mode (Figure 13b) accumulates in the range 0.1–0.14 m owing to the phase matching, then decreases slowly due to the influence of wave diffraction. The calculated RANP shows an approximately linear increase (Figure 13c), which corroborates both the analytical and numerical analyses in Section 2 and Section 3.1.

### 4.2. Development of Linear/Nonlinear Damage Indices

Based on the above analysis in Section 3 and Section 4.1, both the linear and nonlinear features can be extracted to develop *DI*s for the purpose of characterization of HVI-induced pitting damage in the inner shielding layer.

**Figure 14:**
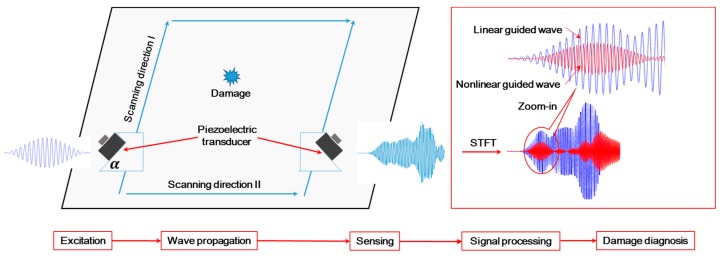
Schematic of linear/nonlinear guided wave-based damage characterization.

A typical GUW-based damage characterization of HVI-induced damage in the inner shielding layer using linear/nonlinear features, as shown in Figure 14, comprises five basic steps, i.e., excitation, wave propagation, sensing, signal processing, and diagnosis. In the excitation step, through a dedicated control of the excitation signal to satisfy both requirements of phase matching and non-zero power flux, the second harmonic wave S_2_ from the case of the mode pair S_1_-S_2_ is accumulated and strengthened for the extraction of linear/nonlinear *DI*s to characterize the HVI-induced pitting damage. The rationale behind the deliberate selection of the S_1_-S_2_ is that usually the probing wave with a higher frequency and higher mode shows a higher sensitivity to damage. Compared to the S_1_-S_2_ mode in the MHz range, the second harmonic in the S_0_-S_0_ mode can only be accumulated with the excitation frequency below 200 kHz (plate thickness of 3 mm), whose low frequency and large time span will cumber the sensitivity and effectiveness of damage characterization.

**Figure 15:**
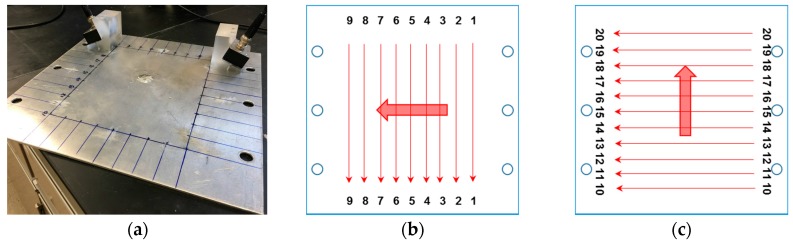
(**a**) Photograph of damage characterization through twenty scanning paths; (**b**) nine vertical paths; and (**c**) eleven horizontal paths.

In the experiment, twenty scanning paths are defined (Figure 15b,c), where the actuator and receiver move in parallel 20 mm at a time (Figure 15a), covering a monitoring area of 160 mm × 200 mm. The excitation of fifteen-cycle Hanning-window modulated sinusoidal toneburst at a central frequency of 1.14 MHz is applied. The phase matching between the mode pair S_1_-S_2_ 3.42–6.84 MHz mm (Figure 13c) leads to the accumulative second harmonic wave S_2_, which is utilized for damage characterization.

**Figure 16:**
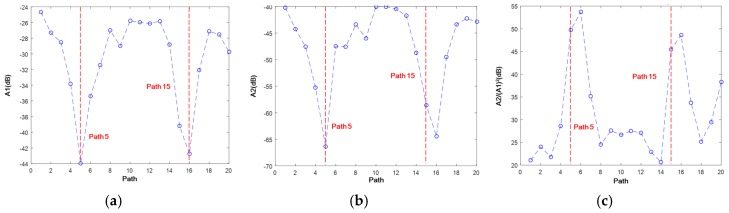
Magnitudes of the mode pair S_1_-S_2_ (3.42–6.84 MHz mm) extracted through STFT on the twenty paths in the experiment in (**a**) fundamental mode A1; (**b**) second harmonic mode A2; and (**c**) A2/(A1)^2^.

In the case of phase matching, S_1_-S_2_ 3.42–6.84 MHz mm, the magnitude of the signal packet is extracted (as illustrated in Figure 14), which indicates the influence of the HVI-induced damage on the linear (fundamental S_1_ mode) and nonlinear (second harmonic S_2_ mode) GUWs (Figure 16). The fundamental probing wave (S_1_) is sensitive to the HVI-induced pitting damage, resulting in a significant energy scattering and reflection upon encountering the damage (a magnitude decrease of 20 dB in Figure 16a). In addition, as the second harmonic wave S_2_ accumulates along the propagation distance, the interaction between S_2_ and damage is manifested via the magnitude of A2 (see Figure 16b), whose decrease of 25 dB indicates a higher sensitivity of the second harmonic mode (S_2_) to damage compared with the fundamental mode (S_1_). The RANP is calculated, (see Figure 16c) highlighting the path with damage. All the three parameters, A1, A2, and RANP, which indicate the existence of HVI-induced pitting damage via different mechanisms, are used as *DI*s.

## 5. Damage Characterization of HVI-Induced Pitting Damage Using Damage Indices

Both linear/nonlinear features of GUWs in the mode pair S_1_-S_2_ of Section 4.2 are utilized in the condition of phase matching, in conjunction with a path-based imaging algorithm, to image the HVI-induced pitting damage.

### 5.1. Construction of Probabilistic Imaging Using the Damage Index

The reconstruction algorithm for the probabilistic inspection of damage (RAPID) [38] is frequently applied in the path-based damage imaging. Inspired by the stimulus of ‘visualizing’ HVI-induced damage, RAPID is performed to project the characterization results to synthetic images. In RAPID, a damage event is manifested according to the probability of its presence, rather than deterministic parameters (damage location, size, shape, etc.). Once an area in the imaging presents a high probability of damage, conventional Nondestructive Evaluation methods can be adopted for a further determination of the damage parameter. In particular, it is almost impossible to use a conventional parameter to define the severity of HVI-induced pitting damage in a large area. Thus, the RAPID, using a probability to describe the HVI-induced pitting damage, indicates its advantage over conventional damage manifestation. In this way, the distribution probability of HVI-induced pitting damage is depicted in a contour map, allowing an intuitive and rapid diagnosis of the inner shielding layer under HVI.

In RAPID, 2-D spatial meshes are firstly built into the inspection area of the inner shielding layer, to which a 2-D pixelated image with each pixel correlated exclusively with a spatial point is mapped. The value of each corresponding image pixel, called the filed value, is able to indicate the presence probability of damage at the spatial point. Thus, an HVI-induced zone with pitting damage can be graphically defined by highlighting those pixels with remarkably high field values as the central region of pitting damage and also noticing some other pixels with relatively small field values as the adjacent region of pitting damage, allowing users to obtain a quantitative and intuitive perception about the HVI-induced pitting damage.

Using the setup of damage characterization in Section 4.2, twenty scanning paths are defined to cover the inspection area. Given a sensing path *P_m_* (*m* = 1, 2, 3, …, 20) with the transducer and receiver at (*y_i_*,*z_i_*) and (*y_j_*,*z_j_*), respectively, the field value at pixel (*y*, *z*) sensed by this sensing path is expressed as

(13) $$\xi {\left(y,z\right)}_{m}=DI_{m}\left[\frac{\varsigma -R_{m}\left(y,z\right)}{\varsigma -1}\right],$$

where *DI* is the defined damage index in the designated path, ς is the scaling parameter controlling the size of the influence area, and *R_m_* is a weight reflected by a distance relationship, which reads,

(14) $$R_{m}\left(y,z\right)=\{\begin{array}{cc} \frac{\sqrt{{\left(y-y_{i}\right)}^{2}+{\left(z-z_{i}\right)}^{2}}+\sqrt{{\left(y-y_{j}\right)}^{2}+{\left(z-z_{j}\right)}^{2}}}{\sqrt{{\left(y_{i}-y_{j}\right)}^{2}+{\left(z_{i}-z_{j}\right)}^{2}}} & when<\varsigma ,\\ \varsigma & when>\varsigma , \end{array}\;,$$

**Figure 17:**
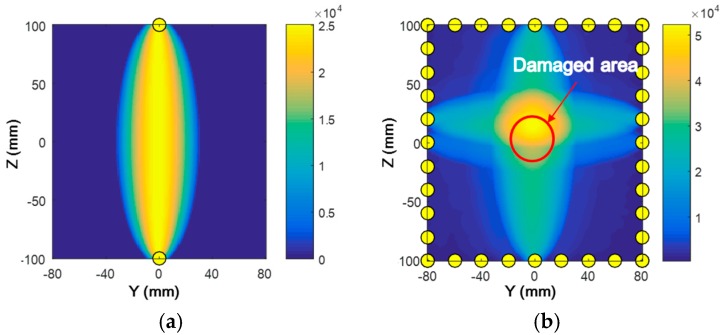

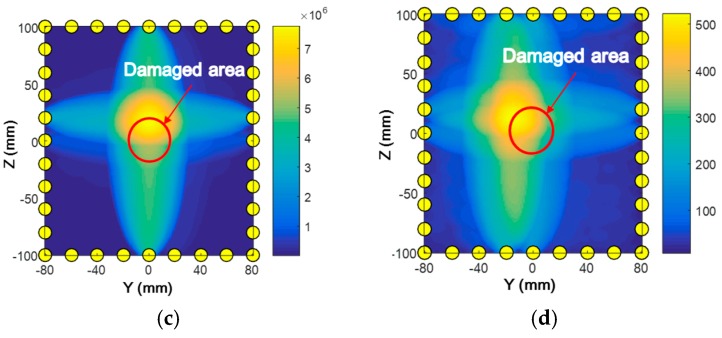
Damage imaging with reconstruction algorithm for the probabilistic inspection of damage (RAPID) in the case of phase matching using various damage indices (*DI*s). (**a**) reciprocal of A1 on path five; (**b**) reciprocal of A1 on all paths; (**c**) reciprocal of A2 on all paths; and (**d**) A2/(A1)^2^ on all paths.

Through Equation (13), the field value at each pixel of the image corresponding to the designated path is obtained, representing the probability distribution of damage, as illustrated in Figure 17a when the reciprocal of A1 on path five is used as the *DI*. Generally speaking, if the inspection area is free of damage, all the influenced pixels in the designated sensing path, for which $R_{m}\left(y,z\right)<\varsigma$ according to Equation (14), show a very low field value owing to the associated *DI* close to zero. On the contrary, if the inspection area is damaged, the field values in the influenced pixels comprise various elliptical loci focused on the two pixels corresponding to the transducer and receiver. The field value gradually decreases as the distance increases for the concerned pixel to the two foci. Once the pixel is outside of the controlled area, $R_{m}\left(y,z\right)=\varsigma$ and accordingly ξ(y,z)=0, the field value is always zero, irrespective of the value of *DI*.

In accordance with Equation (13), any individual sensing path *P_m_* draws a source image, describing the probability as to the HVI-induced damage in the inner shielding layer regarding the single sensing path *P_m_*. Nevertheless, damage may occur distant from most of the paths and can only be sensed by certain paths, and the measuring noise and uncertainty are other concerns if only a sparse path network is introduced. To address both issues, an image fusion scheme is introduced. By amalgamating the available source images of all the sensing paths at the pixel level, an ultimate superposed image is constructed, which renders a collective consensus as to the HVI-induced damage from the entire scanning network. The image fusion scheme, according to the twenty scanning paths in Figure 15, is defined as

(15) $$\xi {\left(y,z\right)}_{sum}=\overset{20}{\underset{j=1}{\sum }}\xi {\left(y,z\right)}_{j},$$

where $\xi {\left(y,z\right)}_{sum}$ is the field value at pixel (*y*, *z*) in the ultimate superposed image. This process of image fusion improves the signal-to-noise ratio, and the damage-incurred singularity stands out compared to the individual source images. Thus, the identified HVI-induced damage zone can be highlighted in the image pixels quantitatively and intuitively.

### 5.2. Imaging: Results and Discussion

The imaging results of HVI-induced pitting damage, using RAPID based on the linear/nonlinear features of GUWs, are presented and discussed. Damage imaging using RAPID based on the linear/nonlinear features in the case of phase matching between the S_1_-S_2_ mode is performed. ς is set as 1.05. As the nonlinear feature—the second harmonic wave—accumulates along the propagation distance, damage can manifest through the magnitude decrease of both A1 and A2. Thus, the reciprocal values of A1 and A2 are taken as the *DI*s for the linear and nonlinear features, respectively, with both imaging results indicating the damage occurrence (Figure 17b,c). The region with the highest pixel value (the orange area) corresponds to the central area of the HVI-induced damage. Considering the form of HVI-induced damage that the central area suffers more severely than the surrounding area, the damage imaging well presents the HVI-induced damage to guide a further repair or replacement. Finally, RANP is also used as the *DI* for damage imaging (Figure 17d), which also highlights the damage location. In addition, one advantage of RAPID over other algorithms is that all the *DI*s for imaging are obtained in the current detected status without the need to acquire any prior knowledge, which improves the applicability of the extracted *DI*s for the characterization of HVI-induced pitting damage.

## 6. Conclusions

The active GUW-based characterization of HVI-engendered damage in the inner shielding layer of spacecraft is pursued. Addressing the understanding of nonlinear second harmonic waves due to various nonlinearity sources, particularly the second harmonic waves, the analytical solution, numerical modeling, and experimental validations are completed and compared, to show their consistency through the analysis on the accumulation of second harmonics under phase matching and phase mis-matching between the fundamental and second harmonic wave modes in both 2/3-D fashions. On the strength of the understanding of all the nonlinear sources, the mode pair S_1_-S_2_ with phase matching is used for the characterization of HVI-induced pitting damage in the inner shielding layer. The linear and nonlinear signal features of guided waves are extracted to calibrate the damage. Compared with the linear components, the nonlinear components feature a larger decrease in amplitude when a sensing path passes the damaged area. Thus, those paths across the pitting damage can be spotlighted using the defined linear and nonlinear *DI*s. Besides, the acquired signals on the paths without crossing damage offer the baseline for the GUW-based damage characterization. In this way, there is no need to record the prior condition, which facilitates the applicability of the currently developed methods. Finally, using *DI*s extracted from both linear/nonlinear features of GUWs, the RAPID algorithm shows the HVI-induced pitting damage to the inner shielding layer.

## Figures and Tables

**Table 1:** Material parameters for the plate structure.

Part	Elastic Modulus (GPa)	Poisson’s Ratio	Density (kg/m^3^)
Plexiglas wedge	4.12	0.39	1180
Aluminum plate	68.9	0.33	2780
	*A* (GPa)	*B* (GPa)	*C* (GPa)
	−320	−200	−190

**Table 2:** Parameters for the mode pairs.

Case No.	Plate Thickness (mm)	Excitation Frequency (kHz)	Frequency Thickness Product (MHz mm)	Mode Pair	Phase Velocity (m/s)
1	1	400	0.4–0.8	S_0_-S_0_	5261–5221
2	3	1178	3.534–7.068	S_1_-S_2_	6241–6241
